# (Fehl‑)Intubation im Rahmen der notfallmäßigen Koniotomie durch den Notarzt

**DOI:** 10.1007/s00101-026-01692-0

**Published:** 2026-05-20

**Authors:** L. Schlattmann, B. Hartung, P. Markwerth

**Affiliations:** Institut für Rechtsmedizin Essen, Universitätsklinikum Essen, Hufelandstraße 55, 45147 Essen, Deutschland

## Einleitung

Fehlintubationen stellen ein relevantes Problem im klinischen Alltag dar, insbesondere im Rahmen der notfallmäßigen Atemwegssicherung und bei schwierigen Atemwegen, bei denen die korrekte Platzierung des Tubus erschwert sein kann. Prädiktoren für einen schwierigen Atemweg können anatomische Gegebenheiten mit Einschränkungen des Bewegungsumfangs als auch pathologische Veränderungen im Gesicht sowie insbesondere in der Mundhöhle sein.

Im Folgenden soll ein Fall einer ungewöhnlichen Fehlintubation im Rahmen einer notfallmäßig durchgeführten Koniotomie vorgestellt werden.

## Falldarstellung

Ein 68-jähriger Mann klingelte aufgrund akuter Atemnot bei seinen Nachbarn. Noch vor Eintreffen des alarmierten Rettungsdienstes kollabierte er auf dem Treppenabsatz des Mehrfamilienhauses und wurde schließlich reanimationspflichtig. Der hinzugerufene Notarzt stellte eine deutliche Zyanose in Gesicht sowie der Zunge fest; die Maskenbeatmung mit einliegendem Guedel-Tubus gestaltete sich als deutlich erschwert. Aufgrund einer einsetzenden Bradykardie mit Frequenzen zwischen 27 und 40/min wurde die Indikation zur Reanimation gestellt. Zudem wurde die Intubation eingeleitet, wobei der Patient, aufgrund von Adipositas und einem gedrungenen Hals, einen erschwerten Intubationssitus aufwies. Im Rahmen der Laryngoskopie konnten eine deutlich vergrößerte Zunge und geschwollene Schleimhäute festgestellt werden. Insgesamt erfolgten drei erfolglose Intubationsversuche, bis der Entschluss zur Durchführung einer Notfallkoniotomie gefasst wurde.

Dem Notarzteinsatzprotokoll ist zu entnehmen, dass eine ca. 2 cm lange Längsinzision zwischen Schild- und Ringknorpel durchgeführt worden sei. Hierdurch sei es zu einer mäßigen, nichtspritzenden Blutung gekommen. Anschließend seien das Lig. conicum ertastet und die Luftröhre mittels Skalpell in Längsrichtung eingestochen worden. Nach Drehung des Skalpells um 90 Grad seien der Eschmann-Katheter und hierüber ein Tubus, Größe 6,0, problemlos eingeführt worden. Es sei zu keiner weiteren, massiven Blutung gekommen. Nach dem Eingriff seien vesikuläre Atemgeräusche über dem linken Lungenflügel festzustellen gewesen, rechtsseitig seien die Atemgeräusche abgeschwächt gewesen. Der Notarzt habe eine Verlegung der Atemwege durch eine Blutaspiration vermutet. Die Messung des exspiratorischen CO_2_ sei positiv verlaufen, wobei lediglich Einzelmessungen und keine kontinuierliche Messung möglich gewesen seien. Der SpO_2_ sei unter suffizienter Reanimation angestiegen. Die Reanimationsmaßnahmen seien unter Etablierung einer Reanimationshilfe (Corpuls CPR, GS Elektromedizinische Geräte G. Stemple GmbH, Kaufering, Deutschland) fortgeführt worden, wobei es zu keinem Zeitpunkt zu einer Wiederherstellung eines Kreislaufes (ROSC) gekommen sei. Nach 47 min seien die Reanimationsmaßnahmen beendet und die Kriminalpolizei bei ungeklärter Todesart verständigt worden.

Bei dem 68 Jahre alt gewordenen Mann waren an Vorerkrankungen Asthma bronchiale, Herzinsuffizienz, Bluthochdruck und Arthrose bekannt. Zudem habe er unter diversen Allergien beziehungsweise Unverträglichkeiten gelitten, insbesondere gegen bestimmte Lebensmittel und Medikamente, wobei sich die konkreten Allergene nicht objektivieren ließen.

Aufgrund von Vorwürfen der Tochter des Verstorbenen gegen den behandelnden Hausarzt und den Notarzt erging durch die zuständige Staatsanwaltschaft der Auftrag zur gerichtlichen Leichenöffnung.

## Wesentliche Sektionsergebnisse

Körperlänge 178 cm, Körpergewicht 93 kg.

Bei der äußeren Leichenschau wurde am Hals, etwa auf Höhe des Kehlkopfes, ein eingebrachter Tubus festgestellt, darüber hinaus angebrachte Defibrillationselektroden und ein Gefäßzugang am rechten Handrücken. Abgesehen von einzelnen bis zu 0,5 cm durchmessenden, narbigen Hautveränderungen an den Beinen lagen äußerlich keine weiteren frischen oder älteren Verletzungen vor.

**Abb. 1:**
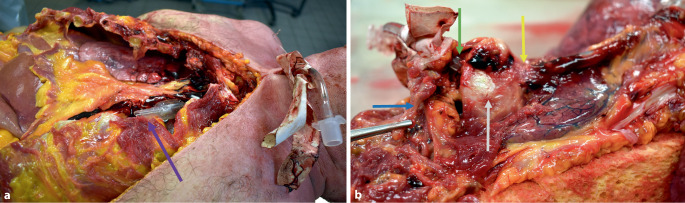
**a** Einblick in den eröffneten Situs. Endlage des Tubus im Weichgewebe zwischen Sternum und Perikard (*violetter Pfeil*). **b** Eintritt (*grüner Pfeil*) des Tubus zwischen Os hyoideum (*blauer Pfeil*) und Cartilago thyroidea (*weißer Pfeil*). Austritt des Tubus unmittelbar unterhalb des Schildknorpels (*gelber Pfeil*)

Die innere Leichenschau erbrachte eine sehr ungewöhnliche Fehllage des durch den Notarzt eingebrachten Tubus. Korrespondierend zu der Hautdurchtrennung mittig am Hals zeigte sich der Tubus unmittelbar oberhalb des Schildknorpels in den Kehlkopf eingeführt, wobei das Lig. thyrohyoideum und die Membrana thyrohyoidea durchtrennt wurden. Der Tubus kam jedoch nicht in der Luftröhre zu liegen, sondern trat unmittelbar unterhalb des Schildknorpels nach vorderseitig aus dem Kehlkopf aus. Die Tubusspitze befand sich im Weichgewebe zwischen Brustbein und Perikard. Das umliegende Gewebe wies ein deutliches Emphysem auf (Abb. 1).

Im Einklang mit der durch den Notarzt geäußerten Verdachtsdiagnose eines allergischen Schocks konnten im Rahmen der Sektion ein deutliches Ödem mit begleitender Rötung der Bronchialschleimhäute, vorwiegend des Kehlkopfeingangs und des Weichgewebes um das Kehlkopfgerüst, festgestellt werden. Die Bestimmung der Mastzelltryptase hätte die Verdachtsdiagnose einer Anaphylaxie unterstützen können, allerdings ist die Aussagekraft insbesondere postmortal eingeschränkt, und ein unauffälliger Wert schließt eine Anaphylaxie nicht sicher aus.

**Abb. 2:**
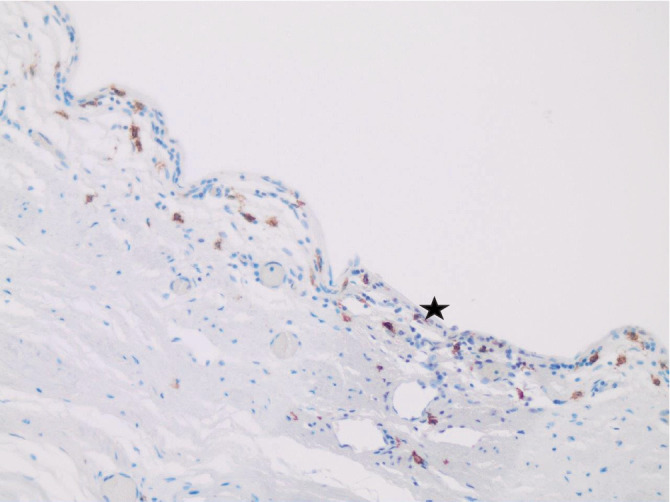
Histologie des Weichgewebes des Kehlkopfes. Färbung der Mastzellen mit CD117 (mit *Asteriskus* markiert), Originalvergrößerung x 100

Im Rahmen der histologischen Untersuchungen konnten Ansammlungen von Lymphozyten insbesondere im Bereich von Larynx und Pharynx, einschließlich des Rachenrings, festgestellt werden. Zum Teil zeigten sich auch eosinophile Granulozyten sowie Ansammlungen von Mastzellen. Letztere wurden insbesondere durch eine Sonderfärbung mit CD117 sichtbar (Abb. 2).

Die Alveolen waren teils kollabiert, größtenteils jedoch, korrespondierend zu dem in der Vorgeschichte bekannt gewordenen Asthma bronchiale, chronisch überbläht.

Dem vorliegenden Notarzteinsatzprotokoll konnte entnommen werden, dass, abgesehen von insgesamt 7 mg Suprarenin und 500 ml Jonosteril, keine weiteren Medikamente verabreicht worden seien. Im Rahmen der toxikologischen Untersuchungen konnten Lidocain, Metoprolol, Enalapril, Diclofenac und Diphenhydramin im Blut festgestellt werden. Die letztendliche Ursache des allergischen Schocks konnte nicht geklärt werden. Prinzipiell wäre eine allergische Reaktion auf eines der eingenommenen Medikamente, insbesondere des Diclofenac, denkbar. Das Lidocain ist aus rechtsmedizinischer Sicht eher auf die notfallmedizinischen Maßnahmen zurückzuführen, wobei dies nicht explizit im Notarzteinsatzprotokoll vermerkt worden ist.

## Diskussion

Der vorliegende Fall beschreibt eine besonders ungewöhnliche Tubusfehllage nach chirurgischer Koniotomie.

Fehlintubationen sind im klinischen Alltag ein häufig vorkommendes Problem und können auch im rechtsmedizinischen Sektionsgut immer wieder festgestellt werden [1]. Besonders in Fällen notfallmäßiger Atemwegssicherung und bei schwierigem Atemweg kann es zu einer Fehlplatzierung des Tubus kommen. In besonders problematischen Fällen liegt eine Situation des „cannot ventilate, cannot oxygenate“ vor, in der weder eine Ventilation noch eine Oxygenierung möglich ist. In derartigen Fällen wird als *Ultima Ratio* die Koniotomie empfohlen [2].

Bei einer Koniotomie handelt es sich um eine reine Notfallmaßnahme zur Atemwegssicherung, die nach Stabilisierung und Hospitalisierung des Patienten, sofern notwendig, durch eine Tracheotomie ersetzt werden muss, falls der Patient nicht auch nichtinvasiv zu beatmen ist [3].

Grundsätzlich kommen zur Durchführung einer Koniotomie unterschiedliche Methoden, im Sinne bspw. eines direkten chirurgischen Zugangs oder der Verwendung eines Trokars, in Betracht. Gemeinsam ist allen Methoden, dass der Zugang durch das Lig. cricothyroideum medianum (Lig. conicum) erfolgt und der Tubus zwischen Schild- und Ringknorpel in die Luftröhre eingeführt wird [4]. Im Vergleich zur Tracheotomie weist die Methode ein geringeres Risiko für Schilddrüsenverletzungen auf, wobei Letztere im klinischen Umfeld als Methode der Wahl anzusehen ist. Hier ist eine Lagekontrolle der Schilddrüse möglich, und eventuelle Blutungen nach Gefäßverletzungen sind besser kontrollierbar [5].

Insgesamt sind Koniotomien grundsätzlich mit einem gewissen, wenn auch eher geringen Verletzungsrisiko verbunden. In der Literatur werden als typische Komplikationen Blutungen, Infektionen, Verletzungen der A. thyroidea, der Schilddrüse, des Ösophagus und des N. laryngeus recurrens genannt [6]. Auch Fehllagen des Tubus werden beschrieben, wobei hier im Wesentlichen Durchstechungen des Kehlkopfes mit Endlage des Tubus im Ösophagus oder Durchstechungen der Tracheahinterwand beobachtet wurden [7–9]. Das Risiko für derartige Fehlplatzierungen wird bei einem chirurgischen Zugangsweg, wie im vorgestellten Fall, als niedriger eingestuft als bei kanülenbasierten Methoden [9]. Der chirurgische Zugangsweg wird grundsätzlich als weniger fehleranfällig angesehen, da die anatomischen Strukturen theoretisch unter Sicht aufgesucht werden können und die Anlage des Tubus hiernach ohne großen Kraftaufwand erfolgen kann. In der Notfallsituation ist diese theoretische Sichtbarkeit jedoch häufig eingeschränkt, insbesondere durch Blutungen im Interventionsfeld sowie erschwerte anatomische Orientierung. Im dargestellten Fall kam es offensichtlich zunächst zu einer Verwechslung der anatomischen Strukturen am Kehlkopf, möglicherweise begünstigt durch den beschriebenen „kurzen Hals“ bei Adipositas. Zudem dürfte auf Grund der drei bereits gescheiterten Versuche, einen nichtinvasiven Zugang zu schaffen, ein erheblicher Zeitdruck aufgetreten sein. Der Tubus muss dann jedoch mit gewissem Kraftaufwand eingeführt worden sein. Hierdurch resultierte eine Fehllage mit mediastinaler Endposition des Tubus, wie sie als bekannte, wenngleich seltene Komplikation im Rahmen notfallmäßiger Atemwegssicherungen vorkommen kann. Im vorliegenden Fall bestand die Besonderheit jedoch nicht allein in der Endlage, sondern in dem der Fehllage zugrunde liegenden atypischen Verlauf mit Durchtritt durch laryngeale Strukturen und anschließender extraanatomischer Passage in das mediastinale Weichgewebe. Somit handelt es sich weniger um eine typische direkte Fehllage, sondern um eine komplexe extraluminale Fehllage im Rahmen der notfallmäßigen Atemwegssicherung. Eine nachträgliche, sekundäre Verlagerung des Tubus in das mediastinale Weichgewebe ist dabei nicht plausibel und wird durch den postmortalen Befund eines kontinuierlichen, extraluminalen Verlaufs nicht gestützt. Im Rahmen der durchgeführten Reanimationsmaßnahmen mittels mechanischer Thoraxkompression (Corpuls CPR) ist ergänzend zu berücksichtigen, dass solche Systeme durch repetitive thorakale Druckbelastung und intrathorakale Druckschwankungen grundsätzlich die Verteilung intrathorakaler Luft sowie die Stabilität von Atemwegshilfen beeinflussen können. Eine relevante nachträgliche Lageveränderung des bereits extraluminal verlaufenden Tubus ist jedoch auch unter diesen Bedingungen nicht anzunehmen. Der vom Notarzt dokumentierte Anstieg der Sauerstoffsättigung (SpO_2_) sowie einzelne positive Kapnometriewerte sind vor diesem Hintergrund kritisch zu interpretieren und lassen sich am ehesten durch Messartefakte, intermittierende Gasverlagerungen im oberen Atemweg sowie die Bedingungen der Reanimation erklären. Ebenso widersprüchlich erscheint im vorliegenden Fall, dass durch den Notarzt angegeben wurde, dass sowohl auskultatorisch Atemgeräusche über den Lungen als auch eine positive Kapnometrie die korrekte Lage des Tubus bestätigt hätten. Die Literatur zeigt jedoch, dass insbesondere die Auskultation nach durchgeführter Intubation insgesamt mit einer hohen Rate an falsch-positiven Ergebnissen einhergeht. So werden in 14 bis 18 % der Fälle, in denen der Tubus im Ösophagus eingelegt wurde, dennoch Atemgeräusche über dem Brustkorb dokumentiert. Die reine Auskultation stellt somit keineswegs immer ein geeignetes Mittel zur Lagekontrolle nach einer Intubation dar [10]. Als Goldstandard ist die kontinuierliche Kapnographie anzusehen, die auch eine Beurteilung der Suffizienz der Reanimationsmaßnahmen ermöglicht [2, 11]. Im dargestellten Fall war eine kontinuierliche Messung jedoch, nach Angaben des Notarztes, nicht möglich.

Vor dem Hintergrund der dargestellten komplexen präklinischen Einsatzbedingungen und der erschwerten Durchführung der invasiven Atemwegssicherung unterstreicht dieser Umstand zugleich die Bedeutung strukturierter Ausbildungskonzepte für invasive Notfalltechniken. Insbesondere praxisorientierte Trainingsprogramme unter Verwendung von Humanpräparaten ermöglichen eine realitätsnahe, haptische Simulation solcher Extremsituationen und können dazu beitragen, die Handlungssicherheit bei Maßnahmen wie der Koniotomie, die im präklinischen Alltag nur in Ausnahmesituationen erforderlich ist, zu verbessern. Entsprechende Ausbildungskonzepte konnten in Studien eine nachhaltige Verbesserung der praktischen Fertigkeiten sowie der Prozedursicherheit zeigen und werden mit einer potenziellen Reduktion schwerer Komplikationen im Rahmen invasiver Atemwegssicherungen in Verbindung gebracht [12, 13].

## Fazit

Fehlintubationen sind ein gängiges Risiko im Rahmen der notfallmäßigen Atemwegssicherung. Die Koniotomie stellt bei Patienten in einer sogenannten Cannot-ventilate-cannot-intubate-Situation eine gute und verhältnismäßig sichere Möglichkeit der Atemwegssicherung dar. Die Maßnahme erfordert ein Aufsuchen und Identifizieren der angestrebten anatomischen Strukturen, wobei diese im Notfallsetting insbesondere durch Blutungen und damit verbundene, eingeschränkte Sichtverhältnisse nur erschwert darstellbar sind. Gerade bei schwer adipösen Patient:innen kann die Notfallkoniotomie zusätzlich technisch anspruchsvoll sein, sodass grundsätzlich immer mit dem Risiko einer Fehllage des Tubus gerechnet werden muss. Eine sichere Lagekontrolle ist daher obligat und kann zuverlässig nur durch den Einsatz der kontinuierlichen Kapnographie erfolgen.

## Data Availability

Die Datensätze, welche in dieser Studie generiert und analysiert wurden, sind auf entsprechende Anfrage von der korrespondierenden Autorin erhältlich.
